# What ails the Pakistani medical institutions?

**DOI:** 10.12669/pjms.314.8575

**Published:** 2015

**Authors:** Shaukat Ali Jawaid

Despite the fact that there is no dearth of talent in Pakistan, our contributions to medical literature and research is very negligible as compared to other Muslim countries like Islamic Republic of Iran, Turkey and even Saudi Arabia. Even our neighboring countries like India and Bangladesh have made tremendous progress during the last two decades but our performance in this field leaves much to be desired. If a critical analysis is made, it will reveal that politicizing of the medial institutions, disregard for merit while making appointments for the heads of various institutions, extensions in service to those with close contact with corrupt politicians and bureaucrats, lack of any faculty development programme, too much political interference in running and managing the medial institutions in the public sector are some of the reasons for this sad state of affairs. As regards the private sector, they are in business, their first priority is to make money hence most of the institutions in the private sector are least interested in research.

An institution is only as good as its faculty but this is one aspect which gets the least attention of our planners and administrators. On the contrary the funds are utilized on buildings and purchase of equipment and other such services which offer lucrative chances for corruption and kickbacks. Little do the authorities concerned realize that availability of costly precious equipment and instruments is of no use if those who are going to use them are not properly trained and experienced? Not only that they are provided facilities to constantly keep themselves update with the latest developments by offering them refresher course and training opportunities.

Yet another important cause of our poor performance is the Pakistani mentality of having “Total Control” leaving very little room for a team work. Just look at the way some of our medical universities are functioning. Their Charter was prepared in such a way that the post of Principal was abolished with the result that the post of Vice Chancellor has been degraded to that of Principal. While the Principal is supposed to look after the day to day affairs of medical college and concentrate on undergraduate medical education, the Vice Chancellor is supped to be a visionary who concentrates on postgraduate studies, training, designing of various postgraduate programmes, faculty development, collaboration with other institutions of higher learning at home and abroad.

At the time of independence, Jinnah Hospital at Karachi and All India Institute of Medical Sciences (AIIMS) at New Delhi started their academic journey together. At present while the AIIMS enjoys international recognition and medial educationists and healthcare professionals consider it an honour to be on the faculty of AIIMS, the JPMC at Karachi has been reduced to a mere district level hospital. It is sad to note that at present out of twenty seven sanctioned posts of Professors, only five are working and the rest about two dozen are vacant. Similar is the situation as regards other faculty poisons i.e. Assistant and Associate Professors.^1^ Hence, what meaningful research one can expect from such an institution which is struggling to provide healthcare to the patients.

Governors being appointed as Chancellor of Medical Universities has also done a great disservice and spoiled these institutions. Let us learn some lessons from our neighboring country Iran where it is the eminent medical personalities with academic accomplishments who are named as the Chancellor of a university. He is assisted by a team of five to six Vice Chancellors each one responsible for Medical Education, Research, Academic Affairs, Services, Patient Care etc. with the result that each one of them concentrates on their area of responsibility and then work as a team for the improvement, betterment of the university. Vice Chancellor responsible for Research takes care of judicious use of allocated research funds, approval of research projects and also makes sure that each research project leads to a publication. According to Prof. Payman Adeebi Vice Chancellor for Research at Isfahan University of Medical Sciences, “research funding is also decentralized. All research proposals are approved by the respective faculties but it is ensured that all research proposals are registered. Funding is provided and research code is also mentioned through the regional Bioethics Committee. Now we have over one thousand research proposals from the postgraduates and faculty every year.” The Deans of various faculties who are provided funding and they allocate it for various research projects and also make sure that these projects are not only completed in time but also result in some publications. A system of accountability is also in place and if there is no publication after the completion of any project because of its poor quality of research, the concerned Dean, Faculty member is held responsible and asked why they approved such a low quality of research work? All this has gone a long way to improve the quality of research work and thus resulting in increased publications.” 2

There are over fifty five medical universities in Islamic Republic of Iran and they all compete with each other in ranking on the basis of research work and publications. The universities also organize an annual Research Day where those faculty members, postgraduates and undergraduates besides other healthcare professionals who have research publications are recognized. The outstanding among them are Awarded Cash Prizes besides other mementoes. The faculty members are also provided all the facilities for their development. Regular workshops on Medical Writing, Research Methodology and Epidemiology are organized. Faculty members are offered incentives on publications in quality standard biomedical journals and those publishing in Impact Fact Journals get additional incentives. The faculty members are also offered financial assistance to attend one medical conference at home and one overseas and all this has played a vital role in promotion of research culture in Iran. Hence at present Tehran University of Medial Sciences itself publishes 31 biomedical journals. Similarly the number medical journals published by Shiraz University of Medial Sciences are 20 and Mashad University of Medial Sciences 17. According to Dr. Ali Akbari Sari Secretary of Commission on Medical Journals in the Health Ministry of Iran, the total number of medical journals published from Islamic Republic of Iran has increased from 91 in 2008 to 347 in 2015. Now they are concentrating on the quality of these journals working to improve them further.^3^

Even in Saudi Arabia, the Government has during the past ten years enormously increased funding for healthcare and research. Numerous medical institutions, universities, Centers of Excellence have been established all over the Kingdom. Medical Universities sponsor large number of research projects. At the Annual Research Day, details regarding publication from last year’s research grants are presented and funding for new projects called as “Signing Ceremony” takes place. Those who excel in research work, with highest number of publications, extra ordinary accomplishments in training, supervising maximum number of postgraduates are honoured and Awarded.^4^,^5^ Hence it is not surprising to note that the contribution of Saudi Arabia to medical literature has seen a significant improvement during the last few years.

In Pakistan Aga Khan University and University of Health Sciences at Lahore which enjoy the First and Second Ranking among the universities in Pakistan by Higher Education Commission have made tremendous progress simply because they respect merit, selection of faculty is merit based and some system of monitoring and accountability exists there. On the contrary in other public sector medical universities, the situation is very bleak. They have not only miserably failed to attract talent but also failed to retain talent whatever they had with the result that we see lot of brain drain. It is not always the financial consideration which prompt talent to fly but when the working environment is not comfortable, the authorities fail to give them due respect and status, patronize and promote mediocres bypassing the talented and experienced faculty just to reward those who have agreed to become permanent members of the “Praise Singers Club”, all this discourages the talent which could otherwise have contributed a lot to the progress and development of these institutions besides contributions to medical literature through research work. Since many countries in the civilized world respect, recognize honour and reward talent, they find easy placements. This is evident from the fact that a large number of faculty members who left Pakistani medical institutions are occupying coveted positions in medical institutions in Saudi Arabia, other Gulf States and even in Malaysia not to forget Europe and North America. The loss of a distinguished researcher and highly respected Medial Editor like Dr. Ahmad Badar who left Pakistan and is currently serving in a university in Saudi Arabia is another classic example of maltreatment of talent in Pakistan. All this has been the gain of these countries but great Loss for Pakistan. Yet another classic example of the way we humiliate and discourage our research scientists is the treatment meted out to Dr. Riazuddin a distinguished research scientist founding Director of CEMB at Punjab University who developed an indigenous economically priced Interferon so that we can manage the epidemic of Hepatitis-C.^6^

At present Pakistan has ninety(90) journals registered with Pakistan Medical & Dental Council. As regards it evaluation criteria for recognition, less said the better. On the contrary Higher Education Commission of Pakistan has a much better evaluation criteria and Indexing system for scientific journals in various categories. So far only eleven scientific journals from Pakistan which includes three medical journals have an Impact Factor as shown in the accompanying table which is one of the important yardsticks to judge the standard and quality of a medical journal. None of the medical journals published by medical universities in Pakistan has so far been able to achieve an Impact Factor which depicts the poor quality as well as quantity of research work undertaken by these institutions.

**Table-I:** Pakistani Scientific Journals with Impact Factor

Rank Full Journal Title		JCR Abbreviated Title	Total Cites	Journal Impact Factor	5-Year Impact Factor
1	Pakistan Journal of Agricultural Sciences	Pak J Agr Sci	473	1.049	Not Available
2	Pakistan Journal of Botany	Pak J Bot	2,913	0.822	0.903
3	International Journal of Pharmacology	Int J Pharmacol	455	0.709	0.652
4	Pakistan Journal of Pharmaceutical Sciences	Pak J Pharm Sci	921	0.682	1.024
5	Journal of Animal and Plant Sciences	J Anim Plant Sci	467	0.448	0.556
6	Pakistan Journal of Zoology	Pak J Zool	628	0.404	0.447
**7**	**JCPSP-Journal of the College of Physicians and Surgeons Pakistan**	**J Coll Physicians Surg Pak**	**812**	**0.353**	**0.393**
8	Journal of the Chemical Society of Pakistan	J Chem Soc Pakistan	656	0.345	0.374
**9**	**Pakistan Journal of Medical Sciences**	**Pak J Med Sci**	**410**	**0.231**	**0.182**
10	Pakistan Journal of Statistics	Pak J Stat	130	0.140	0.258
**11**	Journal of Pakistan Medical Association (JPMA)*	**J Pak Med Assoc**	**1430**	**0.414**	

*Source:* Thomson Reuters WOS Journal Citation Report 2014.

Situation in Pakistan in the field of academics is not going to improve until some radical surgical intervention is undertaken. Some of the measures which can improve the situation and help promote research culture and increase our contribution to world medical literature are as under:

1. Depoliticizing the academic, medical institutions.
2. Appointments of distinguished academicians, researchers, educationists as Chancellor of universities.
3. A team of Vice Chancellors from amongst the faculty should be given responsibilities in different areas one of them being Vice Chancellor for Research with clearly identified responsibilities to look after development work in their respective area.
4. All medical universities must have a post of Principal for their constituent medical or dental colleges to look after the day to day affairs of their respective colleges.
5. Selection of Faculty on merit and merit alone disregarding any other considerations. Further promotions should be liked with their academic accomplishments rather than time scale promotions. “Seniority alone” for promotion is a very fatal disease and all efforts should be made to make sure that institutions do not get infected with this virus.
6. Comfortable working environment for the faculty, giving them due respect which they deserve.
7. Today we are living in an era of professionalism. Hence, the authorities must go for professionals in different departments like information technology, hospital administration, patient safety etc.
8. Statisticians should be appointed in all the medical institutions to help the researchers in sample size selection and data analysis. This is one of the most important problems faced by the faculty while conducting, documenting and publishing research work.
9. Annual clinical audit of every academic department must be made mandatory and it should be published.
10. Distinguished researchers with significant contribution to research should be recognized, honoured and Awarded at the Annual Research Day which will prompt others to undertake research.
11. Faculty Development should be given a serious thought and they should be provided opportunities in the shape of Refresher Courses, Workshops to further enhance and develop their skills.
12. Innovations in medical education should not only be encouraged and facilitated but those involved in such activities should be given special incentives.
13. Judicious use of allocated resources should be ensured.
14. Universities, medical institutions should be allocated funding based on their research output, publications and training of human resources i.e. FCPS, M.Phil, MS, MD, PhD and different other Certificate Courses.
15. Finally no one should ever get an extension in their job. Let everyone play his/her innings and retire honorably rather than begging for extensions in service on one pretext of the other. If they are so keen to serve, they can offer their services in honorary capacity which will be welcomed by the respective institutions.
